# Change of proteolysis and sensory profile during ripening of Cheddar‐style cheese as influenced by a microbial rennet from rice wine

**DOI:** 10.1002/fsn3.1003

**Published:** 2019-03-25

**Authors:** Xiao Zhao, Zhe Zheng, Jian Zhang, Abid Sarwar, Tariq Aziz, Zhennai Yang

**Affiliations:** ^1^ Beijing Advanced Innovation Center for Food Nutrition and Human Health Beijing Technology and Business University Beijing China; ^2^ Beijing Engineering and Technology Research Center of Food Additives Beijing Technology and Business University Beijing China

**Keywords:** Cheddar‐style cheese, microbial rennet, proteolysis, taste profile, volatile compounds

## Abstract

To test the potential of a novel microbial rennet isolated from traditional fermented rice wine for cheese making, Cheddar‐style cheese made with this enzyme was studied for changes in composition, proteolysis, and sensory profile during 90 days of ripening in comparison with a control cheese made with a commercial rennet. The initial proteolysis assay of the microbial rennet on milk proteins indicated a notable increase in the hydrolysis of casein components (α‐, β‐, and κ‐caseins) but no effect on whey proteins upon increasing the concentration of the enzyme. Correspondingly, compared to cheese made with commercial rennet, the use of the microbial rennet in Cheddar‐style cheese resulted in significantly higher primary and secondary proteolysis in the later stages of ripening (60–90 days ripening) and thus a softer texture and the formation of more volatile compounds and free amino acids (FAAs) despite its lower moisture content (41.7%, w/w). Though the cheese made with the microbial rennet was found to contain bitter‐taste FAAs (1,000 mg/100 g), the combined effect of other‐taste FAAs, including sweet (231 mg/100 g), umami (225 mg/100 g), and tasteless (361 mg/100 g) FAAs, in the cheese attenuated the bitter taste of the cheese. This analysis was in accordance with the sensory evaluation, which showed no significantly different sensory scoring between the cheeses made with the microbial and commercial rennets. The present study demonstrated a novel approach to evaluate the bitter taste of ripened cheese. The results of this study suggest the potential of the microbial rennet from rice wine to serve as a new source of milk‐clotting agents in cheese making.

## 1 INTRODUCTION

Milk‐clotting enzymes (MCEs) can be obtained from various sources, including animals, plants, and microbes, and these enzymes play important roles in curdling milk and affecting cheese maturation (El‐Tanboly, El‐Hofi, Youssef, El‐Desoki, & Ismail, 2013). The MCEs of microbial origin have been widely used in making cheeses with advantages, such as wide varieties of microbial sources, rapid growth of microbes, wide biochemical diversity, lower production cost, and easier genetic modification (Lemes et al., 2016). Microbial MCEs can be obtained from *Bacillus* species (Ahmed & Helmy, 2012) or fungi such as *Aspergillus oryzae *(Vishwanatha, Rao, & Singh, 2010).

Microbial MCEs are characterized with generally greater proteolytic activity and different substrate specificity, and these characteristics might intensify cheese ripening (Jiang, Chen, Xue, & Chen, 2007). The proteolytic activity of the MCEs varies with their producing microbes, leading to different extents of proteolysis in cheese during ripening and affecting cheese quality attributes differently (Soltani, Boran, & Hayaloglu, 2016). The use of the rennet from *Bacillus subtilis* K‐26 in Cheddar cheese was shown to cause faster acid development, lower protein and fat contents, and greater soluble nitrogen contents in cheese during ripening (Krishna Rao & Mathur, 1979). The reduced‐fat Cheddar cheese made with the rennet from *Rhizomucor miehei* showed faster proteolysis than cheese made with recombinant camel chymosin. Miniature Cheddar‐type cheese made with the rennet from *B. amyloliquefaciens* had a softer texture and reduced ripening time compared with the cheese made with calf rennet (An, He, Gao, Zhao, & Zhang, 2014). Cheddar cheese made with the rennet from *A. oryzae* had a reduced level of butyric acid, although the maturity index and casein degradation were not significantly affected (Kumar, Sharma, Saharan, & Singh, 2004). However, there was no significant difference in proteolysis during ripening of Prato cheese made with the rennet from *Thermomucor indicae‐seudaticae *N31 compared with cheese made with a commercial coagulant (Merheb‐Dini, Garcia, Penna, Gomes, & Silva, 2012).

The selection of suitable MCE could support the desired modification of yield and flavor with increased ripening and better sensory attributes of cheese. Recently, in the authors’ laboratory, a microbial rennet was isolated from glutinous rice‐fermented wine that was traditionally used for making Chinese Royal Cheese (Zhao, Wang, Zheng, Zhao, & Yang, 2015). The microbial rennet was characterized as having strong milk‐clotting activity and moderate proteolytic activity. The present study was carried out to evaluate the effect of this microbial rennet on proteolysis and sensory attributes of Cheddar‐style cheese, aiming to test its potential for application in cheese making in view of increasing demands for cheese worldwide. The chemical composition, proteolysis, texture, microstructure, volatile compounds, and sensory attributes of the cheese made with the microbial rennet were determined by comparing with those of the cheese made with a commercial rennet. Since the flavoring characteristic of cheese was of particular importance in determining the degree of ripening and consumer acceptance of the product (Ziino, Condurso, Romeo, Giuffrida, & Verzera, 2005), volatile compounds and the bitter taste of cheese formed during ripening of cheese were further analyzed by principal component analysis (PCA) method and evaluated on the basis of taste profiles of free amino acids (FAAs) in cheese.

## 2 MATERIALS AND METHODS

### 2.1 Materials

The starter culture strains, *Lactococcus lactis* subsp. *lactis *XZ3303 and *Lc*. *lactis* subsp. *cremoris *QH27‐1, were obtained from the Inner Mongolia Agricultural University of China and stored in M17 medium containing 70% glycerine at −80°C. The two strains were activated by two consecutive transfers in M17 medium at 30°C and then propagated in milk for use in cheese making at a ratio of 1:1. The commercial rennet (CHY‐MAX Powder Extra NB, 3.17 × 10^5^ Su (Soxhlet units)/g) used for making Cheddar‐style cheese in this study was purchased from Chr. Hansen, Inc. (Khusham, Denmark). The microbial rennet (1.09 × 10^5^ Su/g) was isolated and partially purified from glutinous rice‐fermented wine that had been traditionally produced in southern China for centuries by using a rice starter called Jiuqu that mainly contains yeasts such as *Saccharomyces cerevisiae* (Zhao et al., 2015). The skim milk powder was purchased from Fonterra (New Zealand); α‐lactalbumin (≥85%) and β‐lactoglobulin (≥90%) powders were purchased from Sigma‐Aldrich (USA), which were reconstituted to the required concentration using distilled water. All chemical reagents used in this study were of analytical grade.

### 2.2 Hydrolysis of milk proteins

To determine the proteolytic activity on milk proteins, 0.5 ml of either the microbial rennet from rice wine or the commercial rennet at different concentrations (0, 20, 40, 80, 160, 320, and 640 Su/ml) was added to 5 ml of 10% skim milk (pH 6.77). After 60 min of hydrolysis at 37°C, the reaction was stopped by immediately heating the samples in a water bath at 90°C for five minutes. Then, samples were taken and stored at 4°C for further analysis by sodium dodecyl sulfate–polyacrylamide gel electrophoresis (SDS‐PAGE) as described by Zhao et al. (2015). To further analyze its hydrolytic action on whey protein, 0.1 ml of the microbial rennet from rice wine and the commercial rennet were added to 1 ml 10 mg/ml α‐lactalbumin and 1 ml 10 mg/ml β‐lactoglobulin, respectively, at a rennet dose of 320 Su/mL. Samples were taken for SDS‐PAGE analysis as described above.

### 2.3 Manufacture of Cheddar‐style cheese

Raw cow milk (protein 3.1%, fat 3.7%) obtained from a local dairy farm in the Qinghai Province of China was used to make Cheddar‐style cheese following the standard procedure described by Zhang et al. (2013). The same batch of raw milk (36 L) was used to make two groups of cheese with three parallel samples (6 L raw milk/sample) in each group, Cheese MR and Cheese CR, referring to the cheese made with the microbial rennet and the commercial rennet, respectively. The propagated culture (1.5%, v/v) was added to raw milk homogenously incubated at 33°C for 30 min, and followed by the addition of CaCl_2 _(0.005%, w/v). Rennet was added at a level of 1,500 Su/L milk, and the milk was left for 45 min for coagulation. Curd was cut into cubes (2 cm^3 ^in size) and healed for 10 min without stirring. Then, the curd was cooked at 39°C until the pH reached 6.1–6.2, at which point the whey was drained. After whey drainage, the curd was cheddared until the pH reached 5.4–5.5. The curd was then milled, salted at 2.0% (w/w of the curd), molded, and pressed at 40 pounds overnight. The pressed cheese was vacuum‐packed. Ripening of the cheese was performed at 4°C for 90 days, and samples were taken at days 1, 30, 60, and 90 for analysis as described below.

### 2.4 Determination of cheese yield and composition

The cheese yield was calculated using the following equation: Cheese Yield = Weight of Cheese × 100%/ (Weight of Milk + Starter Culture + Salts). The total protein content of cheese was measured by the Kjeldahl method (AOAC, 1990a, 1990b, 1990c, 1990d), the fat content was measured by the Babcock method (AOAC, 1990a, 1990b, 1990c, 1990d), and the moisture content was determined by oven‐drying at 102°C (AOAC, 1990a, 1990b, 1990c, 1990d). For the pH test, the cheese sample (10 g) was graded and blended in 12 ml of deionized water and then measured by using a calibrated pH meter (FE20 pH meter, Mettler Toledo, Switzerland). The salt content of cheese was determined using the Volhard method (AOAC, 1990a, 1990b, 1990c, 1990d).

### 2.5 Proteolytic parameters

The total nitrogen (TN) content of cheese was determined by the Kjeldahl method (AOAC, 1990a, 1990b, 1990c, 1990d). The water‐soluble extracts (WSE), 70% (w/w) ethanol‐soluble nitrogen (EtOH‐SN), and 5% (w/w) phosphotungstic acid‐soluble nitrogen (PTA‐SN) were prepared according to the method previously reported by Wang, Zheng, Zhao, Yang, and Yang (2015).

### 2.6 Texture profile analysis (TPA)

The TPA was determined by the method described previously by Dabour, Kheadr, Benhamou, Fliss, and LaPointe (2006) with slight modifications. Cheese samples were cut into 15‐mm cubes and maintained at room temperature for 1 hr before testing. The texture parameters of the cheese samples were determined after 1, 30, 60, and 90 days of ripening by performing the texture profile analysis using the TexturePro CT V1.4 Build 17 (Brookfield Engineering Labs, Inc., USA), and the cheese samples were compressed by 50% with the TA11/1,000 compression plate probe by using two compression cycles at a constant compression speed of 0.4 mm/s.

### 2.7 Solid‐phase microextraction/GC‐MS analysis of volatiles

The extracts of volatile compounds were analyzed by solid‐phase microextraction coupled to gas chromatography–mass spectrometry (SPME‐GC‐MS) using the procedure of Wang et al. (2015), and compounds were identified according to NIST 2.0 mass spectrum libraries installed in the GC‐MS equipment.

### 2.8 Assay of free amino acids (FAAs)

The content of FAAs in cheese was determined by a modified method of Diana, Rafecas, Arco, and Quilez (2014) using an HPLC system (Agilent Technologies, USA) under the following conditions: ammonia resin column (2.6 × 150 mm), oven temperature of 53°C, injection volume of 50 μl, flow rate of 0.5 ml/min, mobile phase of citric acid and sodium citrate buffer, and detection wavelengths of 570 and 440 nm.

### 2.9 Sensory evaluation

Sensory characteristics of cheese samples after 90 days of ripening were evaluated by a modified method of Awad, Hassan, and Muthukumarappan (2005), for flavor (sweetness, milkiness, saltiness, bitterness, and acidity), texture (creaminess, elasticity, chewiness, friability, and roughness), and overall preference on a ten‐point hedonic scale (0 = least obvious to 10 = most obvious) for each attribute, by a panel of 10 assessors drawn from faculty members and postgraduate students in the authors laboratory who had been trained for sensory evaluation.

### 2.10 Statistical analysis

The results are presented as the means ±*SD*. All data analysis was performed using spss version 16.0 (SPSS, Inc., Chicago, IL, USA). Significant differences between treatments were tested by ANOVA. All measurements were performed for the two groups of cheese (Cheese MR and Cheese CR) with three parallel cheese samples in each group from two independent trials of cheese making.

## 3 RESULTS AND DISCUSSION

### 3.1 Hydrolysis of milk protein

**Figure 1:**
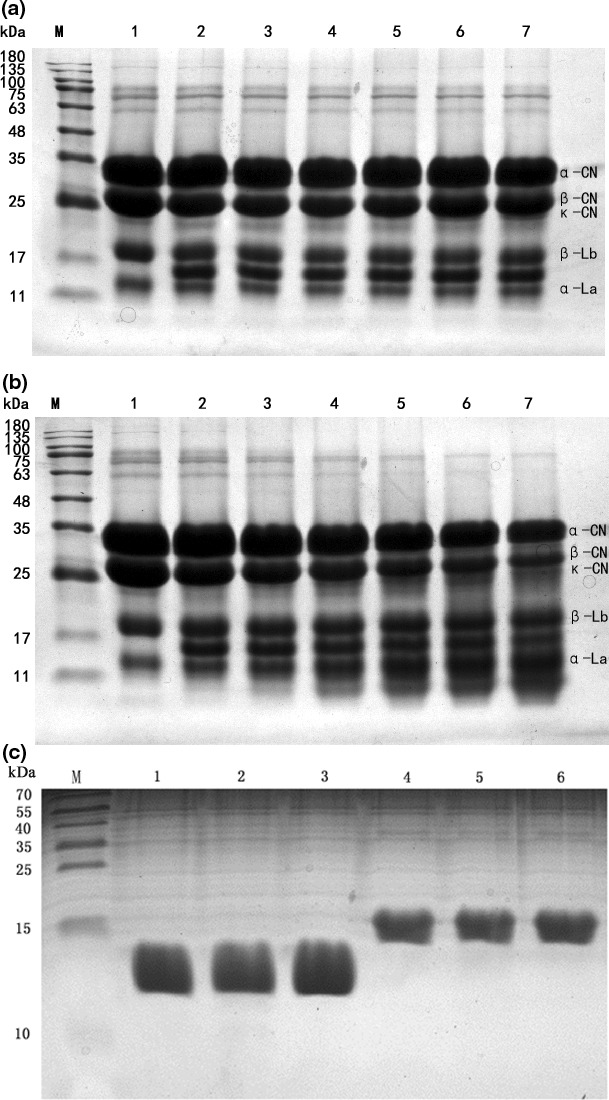
SDS‐PAGE of skim milk showing different protein components hydrolyzed by the commercial rennet (a) and the microbial rennet from rice wine (b) at different concentrations. M: Marker, Lane 1 to lane 7 corresponding to 0, 20, 40, 80, 160, 320, and 640 Su/ml, respectively. (c): SDS‐PAGE of α‐lactalbumin and β‐lactoglobulin without adding rennet (lane 1 and lane 4); α‐lactalbumin and β‐lactoglobulin after degradation by microbial rennet (lane 2 and lane 5); α‐lactalbumin and β‐lactoglobulin after degradation by commercial rennet (lane 3 and lane 6)

The proteolytic activity of the microbial rennet on milk protein components compared with that of the commercial rennet was studied as shown in Figure 1. While the commercial rennet showed no noticeable effect on the major milk protein bands though it hydrolyzed κ‐casein (Figure 1a), the microbial rennet from rice wine could degrade the milk proteins, including α‐, *β*‐, and κ‐caseins (Figure 1b), but not whey protein components, including α‐lactalbumin and *β*‐lactoglobulin (Figure 1c), with increasing concentrations up to 640 Su/ml of the enzymes, confirming the specificity of the microbial rennet to hydrolyze caseins. Similarly, a tamarillo coagulant did not hydrolyze whey protein after 24 hr of treatment (Li, Scott, Otter, Zhou, & Hemar, 2018). The constant peptide band between 11 and 17 kDa in both Figure 1a,b is para‐κ‐casein with a molecular weight of 12,268 Da, as reported by Egito et al. (2007). In Figure 1b, at greater concentrations of the microbial rennet (lane 6 and lane 7), increasing densities of peptide bands lower than 11 kDa and some faint bands at approximately 20 kDa were also observed, probably due to hydrolyzed products from caseins by the microbial rennet. In addition, the microbial rennet seemed to be more effective in degrading κ‐casein than α‐ and β‐casein components since the κ‐casein band faded away at an early stage with a low rennet concentration. Similar proteolytic activity on casein components by the rennet preparation from *B. subtilis *MTCC 10,422 was also reported (Kumari Narwal, Bharat, Ajay, Anil, & Sarla, 2016). The SDS‐PAGE results also indicated a broad specific activity of the microbial rennet from the rice wine on milk caseins, as reported for other microbial proteases, such as an extracellular metalloprotease from the edible mushroom *Termitomyces clypeatus* (Majumder, Banik, & Khowala, 2015) and the milk‐clotting enzyme from *Paenibacillus *spp*.* BD3526 (Hang et al., 2016). The proteolytic pattern of milk proteins by the microbial rennet of this study suggested a possible effect of the enzyme on the cheese quality as a result of its proteolytic activity.

### 3.2 Cheese composition and yield

After 90 days of ripening, Cheese MR had no significant difference (*p* > 0.05) in cheese composition and yield from Cheese CR (Table 1), though slightly greater protein and fat contents (23.18%, 24.68%) were found in Cheese MR than in Cheese CR (21.64%, 23.04%). In addition, slightly lower yield and moisture content (9.60%, 41.70%) in Cheese MR than in Cheese CR (9.87%, 44.13%) were observed. Lower moisture content (37.98%–40.90%) in Cheddar cheese with reduced salts was also reported by Murtaza et al. (2014). Furthermore, the pH values of Cheese MR (pH 5.15) and Cheese CR (pH 5.05) were almost the same.

**Table 1:** Composition of Cheddar‐style cheese

Composition	Cheese MR	Cheese CR
Yield（%,w/w）	9.60^a^ ± 0.22	9.87^a^ ± 0.03
Moisture（%,w/w）	41.70^a^ ± 2.28	44.13^a^ ± 1.09
Protein（%,w/w）	23.18^a^ ± 0.71	21.64^a^ ± 0.46
Fat (%,w/w)	24.68^a^ ± 0.65	23.04^a^ ± 0.33
Salt（%,w/w）	1.31^a^ ± 0.10	1.14^a^ ± 0.04
pH	5.15^a^ ± 0.11	5.05^a^ ± 0.02

Note

Values presented are means ± *SD* of data from triplicate analysis on duplicate trials. a: Means in the same row followed by different superscripts are not significantly different (*p *> 0.05).

### 3.3 Proteolysis

Proteolysis in Cheese MR occurred mainly during the first 30 days of ripening, with only a slight increase from day 30 to day 90, while proteolysis in Cheese CR proceeded slowly during the whole period of ripening. The proteolytic activity of the microbial rennet used in Cheese MR appeared to play the major role in promoting protein degradation during ripening, as observed with many other types of cheese made with microbial rennets (Giannoglou et al., 2016).

**Figure 2:**
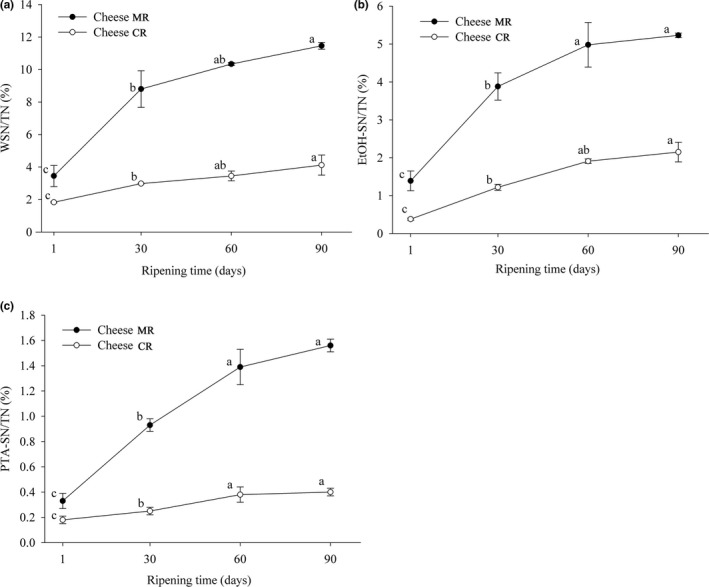
WSN/TN (a), 70%‐ETHANOL‐SN/TN (b), 5% PTA‐SN/TN (c) Changes in the Cheddar‐style cheeses made with microbial rennet from glutinous rice fermentation (Cheese MR) and commercial rennet (Cheese CR) during 90 days of ripening at 4°C. Values presented are means ± *SD* of data from triplicate analysis on duplicate trials. abc Means with different lowercase letters are significantly different (*p* < 0.05) between each day for each type of cheese during the ripening periods

The mean values of WSN, EtOH‐SN, and PTA‐SN are expressed as percentages of TN and are presented in Figure 2a–c, respectively. The WSN representing primary proteolysis in cheese during ripening was found to increase significantly (*p* < 0.05) in each batch of cheese (Figure 2a). The level of primary proteolysis was considerably greater in Cheese MR (from 3.45% to 11.46%) than in Cheese CR (from 1.83% to 4.12%). Similarly, cheese made with the rennet from *B. stearothermophilus* had greater WSN content than cheese made with a commercial coagulant due to additional proteolysis caused by the microbial coagulant (Ahmed, Wehaidy, Ibrahim, Abd El Ghani, & El‐Hofi, 2016). The trend of change with EtOH‐SN during cheese ripening was similar to that with WSN in both cheeses. The EtOH‐SN value of Cheese MR increased from 1.39% to 5.23% and of Cheese CR from 0.38% to 2.15%, and these results were attributed to primary proteolysis (Figure 2b). An et al. (2014) reported a greater TN of pH 4.6‐SN in Cheddar cheese using the rennet from *B. amyloliquefaciens* than in cheese produced with calf rennet. With increasing concentrations of the rennet from *R. miehei*, the pH 4.6‐SN content in reduced‐fat Cheddar cheese also increased significantly (Soodam, Ong, Powell, Kentish, & Gras, 2015).

For the secondary proteolysis during ripening, the PTA‐SN contents in Cheese MR (from 0.33% to 1.56%) were significantly (*p < *0.05) greater than those in Cheese CR (from 0.18% to 0.4%) (Figure 2c). The PTA‐SN fractions were mainly derived from the hydrolysis of peptidase by starter autolysis, representing smaller‐sized peptides and free amino acids. Therefore, the microbial rennet seemed to be capable of hydrolyzing protein to generate more peptides that were in turn used by microbial starters to produce free amino acids. However, the rennet alone also produced a limited range of free amino acids (O'Keeffe, Fox, & Daly, 1976). As both the nitrogen contents of EtOH‐SN and PTA‐SN increased significantly throughout ripening, it was clear that the evolution of nitrogenous substances during cheese ripening was significantly affected by the microbial rennet used.

### 3.4 Texture analysis

Table 2 shows that the values of all textural parameters of Cheese MR and Cheese CR decreased significantly (*p* < 0.05) during the whole ripening period. Comparatively, Cheese MR exhibited more cohesiveness, chewiness, and adhesiveness than Cheese CR (*p* < 0.05) at the beginning of ripening, but no significant difference (*p* > 0.05) was observed in these parameters between the two groups of cheese after 30 days of ripening. Cheese MR and Cheese CR had similar levels of springiness (*p* > 0.05) up to 60 days of ripening, but Cheese MR had lower springiness (*p* < 0.05) after 90 days of ripening. Regarding the hardness of cheese, Cheese MR was significantly (*p* < 0.05) harder than Cheese CR up to 30 days of ripening with an inverted trend of change in hardness of the two groups of cheese during the later period of ripening. The greater hardness, cohesiveness, chewiness, and adhesiveness of Cheese MR during the initial stage of ripening might be due to its lower moisture content (41.70%) and greater salt‐in‐moisture (3.14%) than those (44.13%, 2.58%, respectively) of Cheese CR (Table 1). It was reported that an appropriate moisture content in cheese was important for moisture to bind with the casein network to maintain good cheese texture, and greater moisture content might cause decreased viscosity and hardness of cheese (Hickey et al., 2018). The lower hardness, cohesiveness, springiness, and adhesiveness of Cheese MR than those of Cheese CR approaching the end of ripening period could be mainly attributed to the greater proteolytic activity of the microbial enzyme that degraded milk proteins during ripening. Microbial rennet could affect the structure of casein by influencing the hydrolysis site and hydrolysis degree of protein, leading to a change in the casein network and thus cheese texture (Soltani et al., 2016). Hydrolysis of αs1‐casein and solubilization of colloidal calcium phosphate were found to weaken the protein network and cheese texture during ripening (Diamantino, Beraldo, Sunakozawa, & Penna, 2014). Greater protein hydrolysis was also found to accompany a softer texture in miniature Cheddar‐type cheeses made with a rennet from *B. amyloliquefaciens *(An et al., 2014). Cheese made with *R. miehei* protease was characterized with greater meltability and evident spaces in cheese matrix (Hayaloglu, Karatekin, & Gurkan, 2014). Nevertheless, softer texture and intense flavor of acceleratively ripened Cheddar cheese were sometimes preferred organoleptically (Iwasawa, Suzuki‐Iwashima, Iida, & Shiota, 2014). Decreased adhesiveness of cheese was also considered to be favorable for the separation of cheese from the packing material by consumers (Sołowiej, Cheung, & Li‐Chan, 2014).

**Table 2:** Changes of hardness, cohesiveness, springiness, chewiness, and adhesiveness of the Cheddar‐style cheeses during 90 days of ripening at 4°C

Texture parameter	Ripening time (days)			
	1	30	60	90
Hardness (*N*)				
Cheese MR	17.68^aA^ ± 0.19	10.07^bA^ ± 0.21	6.10^cB^ ± 0.11	5.84^cB^ ± 0.08
Cheese CR	15.69^aB^ ± 0.05	8.97^bB^ ± 0.04	8.47^cA^ ± 0.05	8.15^dA^ ± 0.06
Cohesiveness (mJ)				
Cheese MR	0.75^aA^ ± 0.03	0.51^abB^ ± 0.10	0.40^bA^ ± 0.22	0.23^bA^ ± 0.01
Cheese CR	0.68^aB^ ± 0.02	0.60^abB^ ± 0.01	0.53^bA^ ± 0.03	0.38^cA^ ± 0.06
Springiness (mm)				
Cheese MR	5.79^aA^ ± 0.22	4.74bA ± 0.11	4.46^bA^ ± 0.52	3.08^cB^ ± 0.10
Cheese CR	5.81^aB^ ± 0.01	4.66^bA^ ± 0.08	4.38^bA^ ± 0.54	4.41^bA^ ± 0.16
Chewiness (mJ)				
Cheese MR	78.47^aA^ ± 4.08	21.94^bA^ ± 4.34	18.91^bA^ ± 3.60	23.60^bA^ ± 3.31
Cheese CR	62.01^aB^ ± 3.33	23.50^bA^ ± 2.16	21.21^bA^ ± 3.71	16.83^bA^ ± 3.95
Adhesiveness (mJ)				
Cheese MR	0.31^aA^ ± 0.05	0.56^aA^ ± 0.14	0.36^aA^ ± 0.13	0.29^aA^ ± 0.09
Cheese CR	0.16^aB^ ± 0.06	0.31^aA^ ± 0.18	0.55^aA^ ± 0.12	0.48^aA^ ± 0.23

Note

Values presented are means ± *SD* of data from triplicate analysis on duplicate trials. abc: Means in the same row followed by different superscripts are significantly different (*p* < 0.05). AB: Means in the same column followed by different superscripts are significantly different (*p *< 0.05).

### 3.5 Volatile analysis

**Figure 3:**
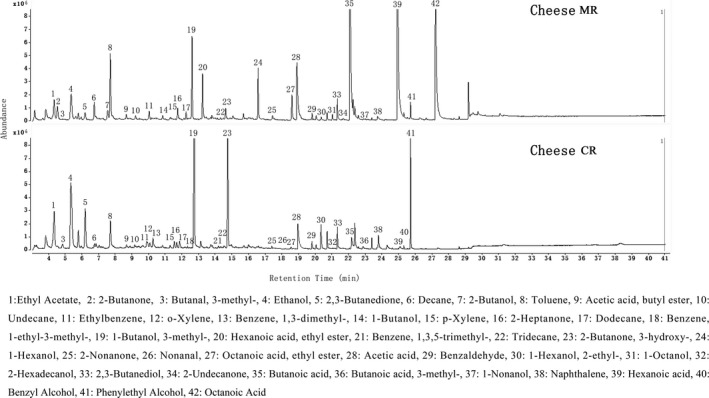
Total ion chromatogram of volatile compounds in Cheddar‐style cheeses made with microbial rennet from glutinous rice fermentation (Cheese MR) and commercial rennet (Cheese CR) after 90 days of ripening at 4°C

The volatile compounds identified in Cheese MR and Cheese CR after 90 days of ripening are shown in Figure 3. A total of 42 volatile compounds, including four alkanes, five acids, six ketones, 10 alcohols, three aldehydes, four esters, nine aromatic compounds, and one unclassified compound, were detected by SPME‐GC‐MS. In Cheese MR, carboxylic acids with low detection thresholds (< 5 mg/kg) (Mei, Guo, Wu, Li, & Yu, 2015), including acetic acid (peak 28) from lactate metabolism, and butanoic acid (peak 35), hexanoic acid (peak 39), and octanoic acid (peak 42) from lipolysis, were most abundant, which were considered to be the key contributors to the aroma profile of Cheese MR. In other types of ripened cheese, carboxylic acids were also found as the major volatile compounds (Ozturkoglu‐Budak et al., 2016). For ethyl esters, four different compounds were detected in Cheese MR, including ethyl acetate (peak 1), acetic acid butyl ester (peak 9), hexanoic acid ethyl ester (peak 20), and octanoic acid ethyl ester (peak 27), and the latter two were considered important odorant compounds in Cheddar cheese (Curioni & Bosset, 2002). These ester compounds contributed to the sweet, floral, and fruity flavors of Cheese MR and helped decrease the rancidity odor derived from carboxylic acids (De Frutos, Sanz, & Martinez‐Castro, 1991). Figure 3 also shows that more volatile compounds were detected in Cheese MR than in Cheese CR, suggesting that the use of the microbial rennet isolated from glutinous rice wine promoted flavor formation in cheese during ripening.

**Figure 4:**
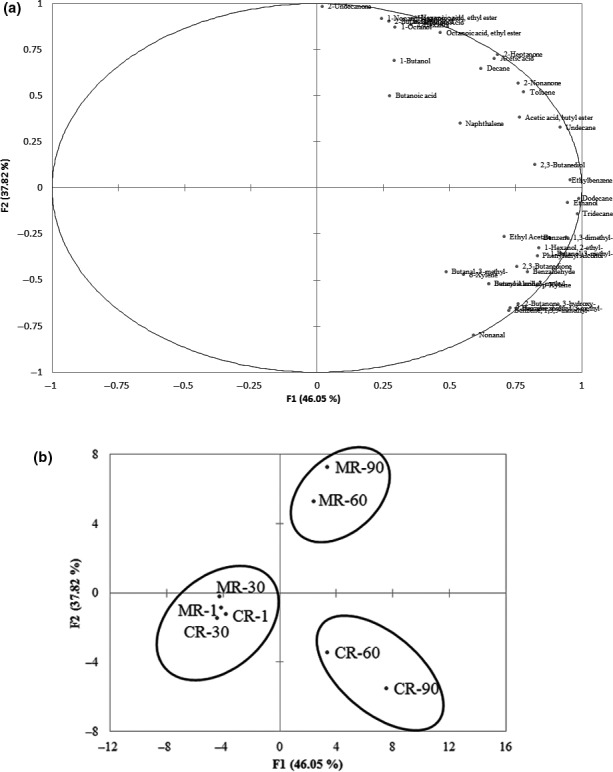
(a) Loading plots after principal component analysis of the variables in the plane defined by the two first principal components (F1 and F2). (b) Scores plot after principal component analysis of the individuals in the plane defined by the two first principal components (F1 and F2). MR: Cheese MR produced with microbial rennet from glutinous rice fermentation; CR: Cheese CR produced with commercial rennet; 1, 30, 60, 90: days of ripening

As flavor formation during ripening was a main factor connected with the maturation and acceptability of Cheddar cheese, principal component analysis (PCA) was performed to determine which were the most important volatile compounds on each day of ripening and to assess how these compounds discriminated between each of the cheeses. Figure 4a shows that all volatiles were on the positive axis of F1 and far from the origin, explaining an important part of the variation. All carboxylic acids (acetic acid, butanoic acid, 3‐methylbutanoic acid, hexanoic acid, and octanoic acid) and most esters (acetic acid butyl ester, hexanoic acid ethyl ester, and octanoic acid ethyl ester) were related to the flavor of cheese at day 90 and were located on the positive axis of F2. This result highlighted the positive correlation with each other and the statistically significant interaction between these compounds, particularly in Cheese MR with greater content of carboxylic acids and esters (Figure 3), indicating beneficial effect of the microbial rennet on volatile formation in Cheese MR. Aldehydes (butanal, 3‐methyl‐, nonanal, benzaldehyde) were located in the lower part of the plane in the negative axis, confirming their flavor defects in both cheeses but less significant effect on Cheese MR for their upper location of the plane at day 60 and day 90 (Figure 4b). Other volatile compounds, such as alkanes, ketones, and alcohols, were located on the plane separately, indicating the discriminated variation of these compounds because of their greater degree of separation in both cheeses.

Figure 4b shows that Cheese MR and Cheese CR were clearly separated by the distribution of the scores on the first two principal components with 3 separate groups of points, corresponding to the different days of ripening. At the beginning of ripening (day 1 and day 30), both cheeses were positioned closely in the negative part of PC1 and PC2, indicating no significant difference between volatile profiles of the two cheeses during the first month of ripening. Both Cheese MR and Cheese CR at day 60 and day 90 were located in the positive area of F1; thus, F1 might be representative of the ripening time. However, Cheese MR with 60 and 90 days of ripening was in the upper part of F2 and Cheese CR in the low part of the plane, indicating different volatile profiles formed between the two cheeses during the third month of ripening. In addition, the close proximity of day 60 and day 90 in Cheese MR confirmed that similar volatile profiles formed between these days of ripening, indicating acceleration of cheese ripening caused by the microbial rennet used. The reduced ripening time of Cheddar cheese caused by using other microbial rennets was also pointed out by An et al. (2014).

### 3.6 Sensory evaluation

**Figure 5:**
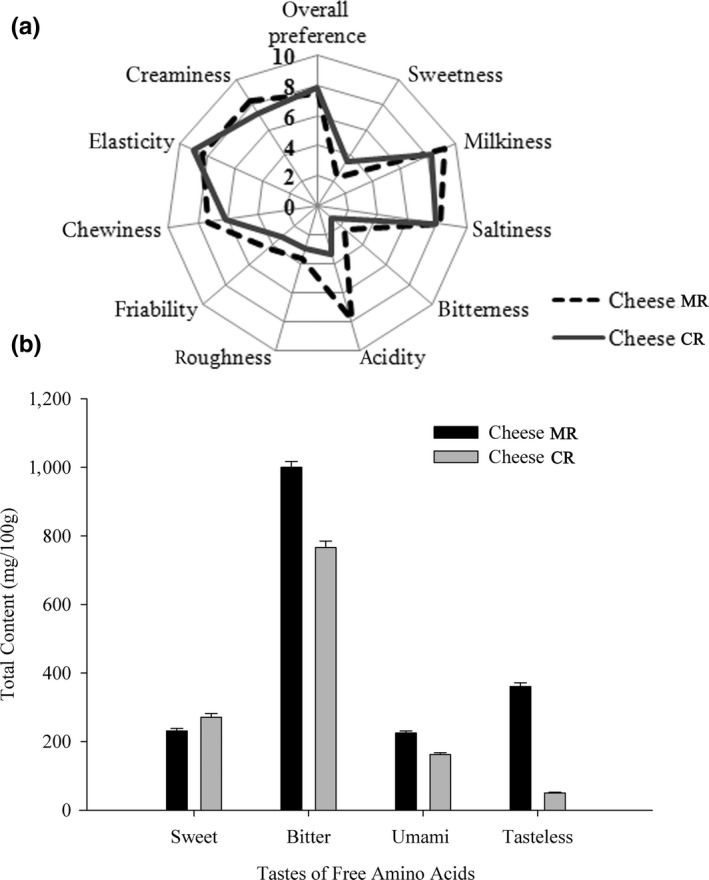
(a) Sensory profile of the Cheddar‐style cheeses produced with microbial rennet from glutinous rice fermentation (Cheese MR) and commercial rennet (Cheese CR) at ripening time. (b) The contents of different tastes free amino acids components in Cheddar‐style cheeses produced with microbial rennet from glutinous rice fermentation (Cheese MR) and commercial rennet (Cheese CR) after 90 days of ripening. Values presented are means ± *SD* of data from triplicate analysis on duplicate trials. Sweet: Thr + Ser +Gly + Ala + Pro; Bitter: Val + Met +Ile + Leu +Arg + Phe +His + Trp; Umami: Asp + Glu; Tasteless: Lys + Tyr

To confirm the acceptability of Cheddar‐style cheese made with MCE from glutinous rice wine, the sensory properties of Cheese MR and Cheese CR after 90 days of ripening were evaluated regarding cheese texture (creaminess, elasticity, chewiness, friability, and roughness), flavor (sweetness, milkiness, saltiness, bitterness, and acidity), and overall preference. As shown in Figure 5a, Cheese MR had no significant difference from Cheese CR in terms of overall preference, and both cheeses showed good acceptability. In particular, no notable bitterness was detected in Cheese MR though there was more proteolysis taking place in Cheese MR than Cheese CR during ripening, as described above (Figure 2). Previously, the rennets from *B. stearothermophilus *and fungi were shown to cause bitter‐taste defects in different cheeses (Ahmed et al., 2016; Vishwanatha et al., 2010). The formation of hydrophobic peptides due to the proteolytic activity of the microbial rennet was thought to cause a bitter taste in cheese (Pino, Prados, Galán, McSweeney, & Fernández‐Salguero, 2009). However, Cheddar cheese made with a microbial (*Aspergillus niger*) rennet possessed better texture and flavor than that made with a commercial calf rennet (Sathya, Pradeep, Angayarkanni, & Palaniswamy, 2012).

Free amino acids (FAAs) as precursors of flavor compounds generated through primary and secondary proteolysis during cheese ripening were thought to contribute significantly to the final taste of cheese (Irigoyen, Ortigosa, Juansaras, Oneca, & Torre, 2007). These FAAs could be classified into four classes as follows: sweet (Thr, Ser, Gly, Ala, Pro), bitter (Val, Met, Ile, Leu, Arg, Phe, His, Trp), umami (Asp, Glu), and tasteless (Lys, Tyr) (Mau, Lin, Ma, & Song, 2001). Figure 5b shows the effect of FAAs formed during ripening of Cheese MR and Cheese CR on the sensory characteristics of cheese, and a considerable difference in the taste profiles of FAAs between the two cheeses was observed. The content of bitter‐taste FAAs (1,000 mg/100 g) was greater in Cheese MR, but the combined effect of other‐taste FAAs such as sweet (231 mg/100 g), umami (225 mg/100 g), and tasteless FAAs (361 mg/100 g) in Cheese MR played important roles in attenuating the bitter taste of cheese. The high level of umami FAAs in Cheese MR (162 mg/100 g) might increase the intensity of other tastes, thus improving the cheese taste (Jinap & Hajeb, 2010). The umami taste, which is indicative of aged Cheddar cheeses, generally increased along with cheese ripening and facilitated cheese flavor positively (Drake et al., 2007). These factors might explain why Cheese MR, containing relatively greater levels of bitter FAAs, did not show a notable bitter taste. A limited level of bitterness in food was sometimes considered to be desirable for variable taste reception in human beings (Meyerhof et al., 2010).

## 4 CONCLUSIONS

In this study, the microbial rennet isolated from glutinous rice wine traditionally used in making Chinese Royal Cheese was tested for its application in Cheddar‐style cheese and compared to a commercial rennet. The results showed that the use of the microbial rennet resulted in Cheddar‐style cheese with lower moisture (41.7%, w/w) and higher levels of primary and secondary proteolysis over a 90‐day ripening period at 4°C. Despite its lower moisture content, the cheese made with microbial rennet was softer and higher concentrations of volatile compounds after ripening for 60–90 days. Analysis of the taste profile of FAAs showed that Cheese MR contained a relatively high level of bitter FAAs, but the combined effects of sweet, umami, and tasteless FAAs in cheese played important roles in attenuating the bitter taste contributed by the bitter FAAs.

Sensory evaluation of cheese confirmed good acceptability with no clear bitter taste in the cheese made with the microbial rennet. Therefore, the microbial rennet isolated from glutinous rice wine in the present study may serve as a new source of milk‐clotting agents in manufacturing cheeses with accelerated ripening properties. Further investigation will be conducted on the functional aspect of the cheese made with the microbial rennet considering the possible bioactive protein or peptide formation during cheese ripening and the application of the microbial rennet in other types of cheeses.

## CONFLICT OF INTEREST

The authors declared that they do not have any conflicts of interest.

## ETHICAL STATEMENTS

This study does not involve any human or animal testing.
